# DNA Barcoding and Molecular Phylogeny of *Drosophila lini* and Its Sibling Species

**DOI:** 10.1155/2012/329434

**Published:** 2012-02-08

**Authors:** Yi-Feng Li, Shuo-Yang Wen, Kuniko Kawai, Jian-Jun Gao, Yao-Guang Hu, Ryoko Segawa, Masanori J. Toda

**Affiliations:** ^1^Department of Entomology, South China Agricultural University, Guangzhou 510642, China; ^2^Institute of Low Temperature Science, Hokkaido University, Sapporo, Hokkaido 060-0819, Japan; ^3^Laboratory for Conservation and Utilization of Bio-resources, Yunnan University, Kunming 650091, China; ^4^Evolutionary Genetics Laboratory, Department of Biology, Tokyo Metropolitan University, Tokyo 192-03, Japan; ^5^The Hokkaido University Museum, Hokkaido University, Sapporo 060-0810, Japan

## Abstract

*Drosophila lini* and its two sibling species, *D. ohnishii* and *D. ogumai*, are hardly distinguishable from one another in morphology. These species are more or less reproductively isolated. The mitochondrial *ND2* and *COI-COII* and the nuclear ITS1-ITS2 regions were sequenced to seek for the possibility of DNA barcoding and to reconstruct the phylogeny of them. The character-based approach for DNA barcoding detected some diagnostic nucleotides only for monophyletic *D. ogumai*, but no informative sites for the other two very closely species, *D. lini *and *D. ohnishii*, of which strains intermingled in the molecular phylogenetic trees. Thus, this study provides another case of limited applicability of DNA barcoding in species delineation, as in other cases of related *Drosophila* species. The molecular phylogenetic tree inferred from the concatenated sequences strongly supported the monophyly of the cluster of the three species, that is, the *lini* clade. We propose some hypotheses of evolutionary events in this clade.

## 1. Introduction

Studies of just diverging populations or species shed light on speciation mechanisms. An important evolutionary process in speciation is the diversification of genes between populations. Most comprehensive information on gene (DNA) evolution associated with speciation has been accumulated for the *Drosophila melanogaster* species subgroup, especially the *D*. *simulans* clade (e.g., [1–6]), and the *D*. *obscura* species group [7, 8]. In comparison, speciation mechanisms have been less explored in the *D*.* montium* species subgroup, in spite of its highest species diversity (= 89) [9] in the *melanogaster* group, with a variety of species at different stages of speciation process. On the other hand, molecular markers have been used to detect cryptic species under incipient speciation process. In the *D*.* montium* subgroup as well, such a molecular approach has recently been employed to reveal the presence of a cryptic species in the *D. serrata* species complex [10]. “DNA barcoding” is proposed as a promising tool not only for rapid identification of known species, that is, “species identification,” but also for discovery and delimitation of species, that is, “species discovery” or “DNA taxonomy” [11–13].

Discovery of a sibling species of *Drosophila lini* is one of cases in which molecular characters were used for “species discovery” in early days. It was first recognized as a species closely related to but different from *D*. *lini* based on the results of electrophoretic analyses [14]. Then, the studied “*D. lini*-like” strain, MMY326, from Pyinoolwin in central Myanmar, along with another strain (MMY307) from the same locality, was described as *D*. *ohnishii* [15]. At the same time, another sibling species, *D*. *ogumai*, was described for two strains (RGN3 and RGN206) from southern Myanmar [15]. It is, however, hard to morphologically distinguish among the three species, *D*. *lini*, *D*. *ohnishii,* and *D*. *ogumai*, especially between the former two, although 80–100% correct classification was achieved for them by discriminant analyses using 13 or 15 quantitative characters [15]. The evidence from cross-tests supports the presence of three sibling species. More or less strong postmating isolation is present among them: no F1 hybrids could be obtained from crosses between *D. ogumai* and *D. ohnishii*, while the other interspecific crosses produced fertile hybrid females but sterile males [16]. In addition, strong premating isolation was detected between *D. ohnishii *and *D. lini *or between *D. ohnishii *and *D. ogumai*, but not between *D. lini *and *D. ogumai *[16–18]. When the *D. kikkawai *species complex was established in the *D. montium *species subgroup of the *D. melanogaster *species group, *D. lini *was included in it [19]. Subsequent molecular phylogenetic studies consistently supported the close relationships between *D. lini *(and its siblings) and *D. kikkawai *(and its siblings, *D. bocki *and *D. leontia*) [14, 20–24]. However, the relationships between *D. lini *and its siblings have not been resolved yet.

Up to date, it is known that *D. ohnishii* is distributed in central Myanmar to southwestern China (Xishuangbanna), just occupying the intermediate range between the ranges of the two allopatric species, *D. lini* distributed in southern China to Taiwan and *D. ogumai* in southern Myanmar [18]. Thus, the premating isolation is seen between the parapatric neighbors of the three species. Based on this biogeographical evidence, a hypothesis that the premating isolation has evolved through the process of reinforcement in the secondary contact zone between *D*. *ohnishii* and either neighboring species has been proposed [18]. To test or refine this hypothesis, the present study aims at revealing reliable phylogenetic relationships among these three species based on DNA sequence data. In addition, molecular diagnostics are searched to apply the DNA barcoding “species identification” to these sibling species that are hard to be distinguished morphologically from one another.

When focusing on very closely related species, one should select rapidly evolving regions, for example, mitochondrial genes [25] or nuclear rDNA internal transcribed spacer (ITS) [26], as markers. The mitochondrial cytochrome *c* oxidase subunit I (*COI*) gene has been widely used as DNA barcoding for “species identification”: its 648-base pair (bp) fragment is the standard marker in the Barcode of Life project [11, 12]. In the present study, we employed two mitochondrial loci, NADH dehydrogenase subunit 2 (*ND2*) and cytochrome *c* oxidase subunit I and II (*COI-COII*), and one nuclear locus, rDNA internal transcribed spacer 1 and 2 (ITS1-ITS2), to examine the phylogenetic relationships among isofemale strains of *D. lini *and its sibling species and to find possible molecular diagnostics for each species of them.

## 2. Materials and Methods

Seven isofemale strains of *D. lini*, four strains of *D. ohnishii,* and two strains of *D. ogumai *were used as focal OTUs, and one strain each of *D. kikkawai*, *D. bocki*, *D. leontia,* and *D. barbarae* of the *kikkawai *complex, and one strain each of *D. jambulina* and *D. seguyi *belonging to the *montium* subgroup were added as ingroup OTUs (Table 1). Three of these isofemale strains (MLN24 and MLN45 of *D. ohnishii* and MLN260 of *D. barbarae*) were established in 2003 from Menglun, southern part of Yunnan Province, China, but all the others derived from the stocks of Tokyo Metropolitan University and have been maintained in laboratory on cornmeal-malt medium at 23°C under continuous light for more than 12 years. The species status of the closely related species, that is, *D. lini*/*D. ohnishii*/*D. ogumai *and *D. kikkawai*/*D. bocki*/*D. leontia*, was confirmed by cross-tests in previous studies [16, 27].

Total DNA was extracted from a single fly using a rapid method [28]. The target regions (Table 2) were amplified on an iCycler Thermal Cycler (Bio-Rad) with the PCR cycle program comprised a 5 min of predenaturation at 94°C, 35 cycles of amplification (1 min of denaturing at 94°C; 1 min of annealing at 53°C for *COI-II* and *ND2*, 56°C for ITS1-ITS2, 1 min of extension at 72°C), and final extension at 72°C for 5 min. The amplicons were purified by precipitation with isopropanol and then subjected to sequencing reaction using BigDye Terminator v3.1 Cycle Sequencing Kit (Applied Biosystems) following the recommended protocol. The sequences were analyzed on the 3100-Avant Genetic Analyzer (Applied Biosystems).

The ITS1-ITS2 sequences of three species of the *montium* subgroup were downloaded from GenBank, AY278412 for *D. barbarae*; AY278419 for *D. jambulina,* and AY278431 for *D. seguyi*. For the three species of the *D. melanogaster *subgroup employed as outgroups, the corresponding sequences were also downloaded from GenBank: AF200829 for *ND2 *and *COI-COII* and M21017 for ITS1-ITS2 of* D. melanogaster*; AF200846 for *ND2 *and *COI-COII* and Z28413 for ITS1-ITS2 of* D. simulans*; AF200831 for *ND2 *and *COI-COII*, Z28538 for ITS1-ITS2 of* D. mauritiana*.

DNA sequences were edited and analyzed using MEGA 5.05 [29]. Phylogenetic trees were constructed by the Neighbor-Joining (NJ) method with bootstrap test (1000 replicates) using the Kimura 2-parameter model, with gaps treated by pairwise deletion. For searching DNA barcoding diagnostics, we focused only on the three sibling species with multiple test strains, and applied both of tree- and character-based methods to each of different loci separately. We used the phylogeny-based approach in the former method, examining the monophyly of each species on a phylogenetic tree [30–33]. The character-based method identifies a set of diagnostic nucleotides in the DNA barcode sequence: the four standard nucleotides (A, T, C, G) if found in fixed states in one species can be used as simple pure diagnostics for identifying that species [34]. To examine molecular genealogies for the focal OTUs, we constructed an NJ tree based on the concatenated sequences of the three loci, and applied an estimated divergence time, 5.4 million years ago (Mya) [35], between *D. melanogaster *and *D. simulans *as a calibration point to estimate the divergence time of each node. Before the analysis using the concatenated sequence data, we conducted a Bayesian concordance analysis to test the concordance among the three regions, that is, *ND2*, *COI-COII*, and ITS1-ITS2, using BUCKy [36]. The DNA sequences of each region were analyzed using MrBayes 3.1.2 [37] for Bayesian phylogenetic estimation. Firstly, phylogenetic trees were constructed for each region via the Markov chain Monte Carlo (MCMC) method (number of generations for runs = 1,000,000, nucleotide substitution model = GTR (general time-reversible)), and then, the output of MrBayes was summarized using the mbsum program of BUCKy, and the primary concordance tree was generated with sample-wide concordance factors using default setting in BUCKy.

## 3. Results

### 3.1. DNA Barcoding for *D. lini* and Its Sibling Species

**Figure 1:**
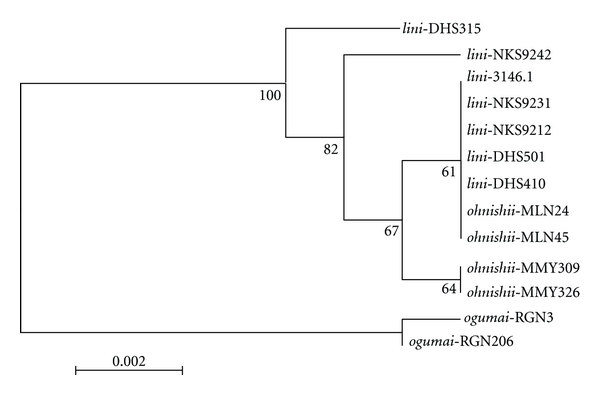
Neighbor-joining (NJ) tree inferred from *ND2* sequences of 13 strains of *D. lini *and its sibling species (*D. ohnishii *and *D. ogumai*). Numbers below branches indicate the bootstrap percentages.

We sequenced the *ND2* gene in *D. lini* and its siblings and some other species of the *montium* subgroup. The whole sequence of this gene is 1206 bp in most species of the *D. obscura* species group [38]. Our obtained sequences covered most of this region (from the site 34 to 959). The alignment of the sequences included no indel. The GenBank accession numbers of these sequences are AY739939-AY739956. The NJ tree for 13 strains of *D. lini *and its siblings showed that *D. ogumai* was monophyletic but that *D. lini *and *D. ohnishii *were nonmonophyletic with overlap of strains of these two species (Figure 1). There were 20 informative sites in the aligned 13 sequences of* D. lini *and its siblings. Of these sites, 11 nucleotides were specific to *D. ogumai*, and thus can be used as diagnostic nucleotides for identification of this species among the siblings (Table 3). However, there was no species-specific, fixed nucleotide for either *D. lini *or *D. ohnishii*.

**Figure 2:**
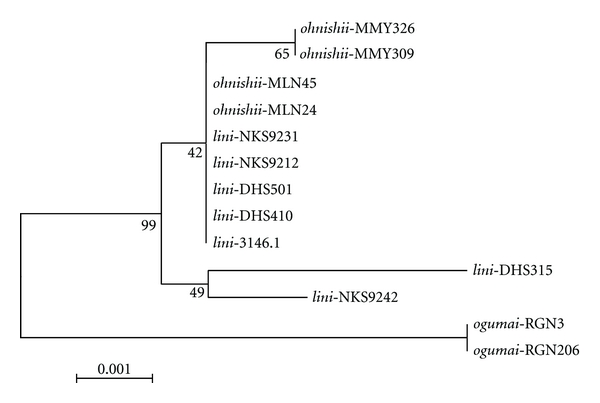
Neighbor-joining (NJ) tree inferred from *COI-COII* sequences of 13 strains of *D. lini *and its sibling species (*D. ohnishii, *and *D. ogumai*). Numbers below branches indicate the bootstrap percentages.

The whole *COI* and *COII *sequences are 1536 and 684 bp, respectively, in *D. yakuba *[39]. The *COI-COII* region we sequenced covered 130 bp of *COI* and 639 bp of *COII*. The GenBank accession numbers of these sequences are AY737604-AY737622. The NJ tree based on the *COI-COII* sequences of the 13 strains of* D. lini *and its siblings showed the monophyly of *D. ogumai* but nonmonophyly for either *D. lini *or* D. ohnishii* (Figure 2). Twelve informative sites were detected from this region, among which five were species-specific, diagnostic nucleotides for* D. ogumai* (Table 4). The character-based approach failed to distinguish between the two non-monophyletic species for the *COI-COII* sequences as well.

**Figure 3:**
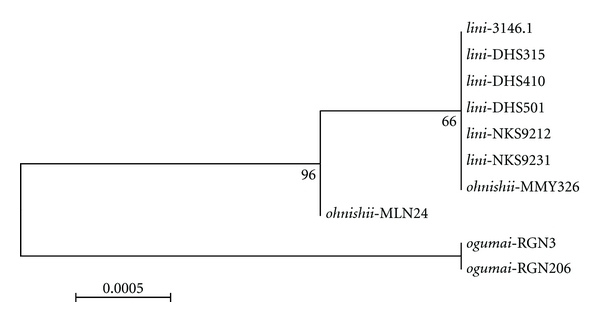
Neighbor-joining (NJ) tree inferred from ITS1-ITS2 sequences of ten strains of *D. lini *and its sibling species (*D. ohnishii *and *D. ogumai*). Numbers below branches indicate the bootstrap percentages.

Sequences of the ITS region covering a part of ITS1, the whole 5.8S rDNA, ITS2a, 2S rDNA, and a part of ITS2 were amplified from 10 strains of *D. lini *and its siblings and some other species of the *montium* subgroup. The positions of nucleotides in the sequence were determined by alignment with the ITS sequence of *D. simulans* [26]. The GenBank accession numbers for these sequences are AY739939-AY739956. The 5.8S rDNA, ITS2a, and 2S rDNA were very conservative in all compared species, without variation in the sequence length. On the other hand, the ITS1 and ITS2 diverged largely in respect of either nucleotide substitution or sequence length. The NJ tree for the 10 strains of *D. lini *and its siblings showed the monophyly of *D. ogumai* but non-monophyly for either* D. lini *or *D. ohnishii *(Figure 3). Four informative sites were present in this region, of which two nucleotide substitutions and one insertion were diagnostic for *D. ogumai* (Table 5); the remaining one, a 14-bp indel (sites 312–325) of ITS2, was polymorphic in *D. ohnishii*. For this region as well, no diagnostic nucleotide was found in either *D. lini *or *D. ohnishii*.

### 3.2. Molecular Phylogeny

**Figure 4:**
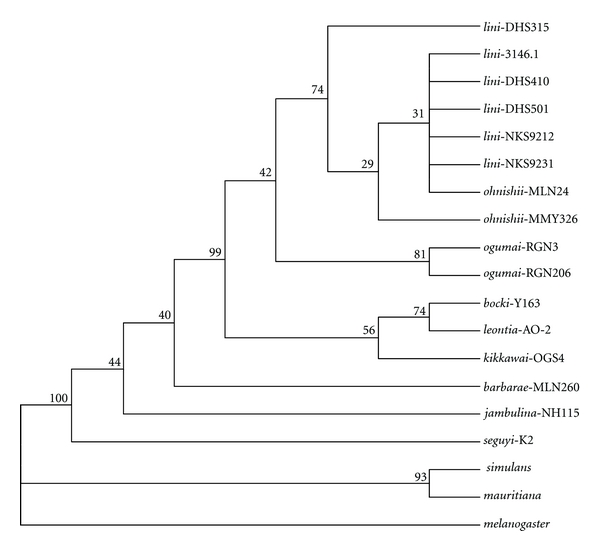
The primary concordance tree resulting from the Bayesian concordance analysis for the three regions, *ND2*, *COI-COII*, and ITS1-ITS2. Numbers above branches are the concordance factors.

**Figure 5:**
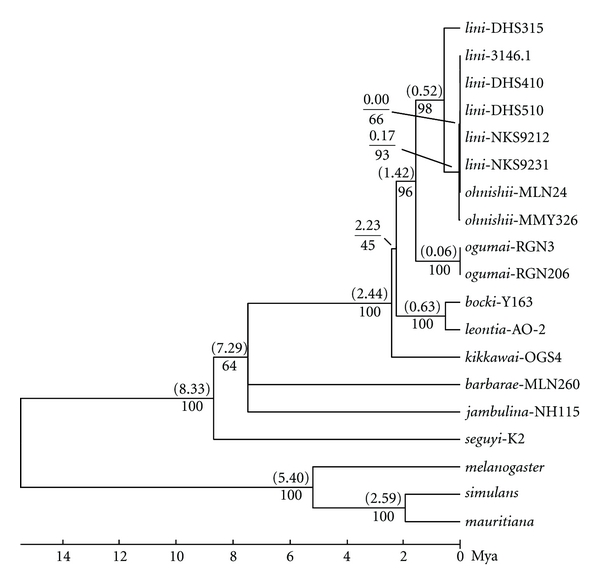
NJ tree inferred from the concatenated sequences (2442 bp) of three regions, *ND2* (926 bp), *COI-COII* (842 bp), and ITS1-ITS2 (674 bp). The time scale (in Mya) was given to the tree on the basis of an estimated time, 5.4 Mya, for the divergence between *D. melanogaster *and *D. simulans* [35] as a calibration point. MEGA 5.05 [29] was used for constructing the tree (bootstrap test: 1000 replications; model: Kimura 2-parameter; gaps: treated by pairwise deletion). Numbers in parentheses above branches indicate divergence times (Mya), and those below branches bootstrap percentages.

The primary concordance tree (Figure 4) resulting from the Bayesian concordance analysis for the three loci (*ND2*, *COI-COII*, and ITS1-ITS2) was not discordant, especially the same for the strains of *D*. *lini *and its sibling species, in topology from the NJ tree (Figure 5) constructed using the concatenated sequences of the three regions (*ND2* + *COI*-*COII* + ITS1-ITS2, 2442 bp in length), indicating that the mitochondrial and nuclear loci are concordant in the genealogies. The estimated divergence times based on a calibration point of 5.4 Mya divergence between *D. melanogaster* and *D. simulans* [35] were also shown in Figure 5. The three focal sibling species, *D*. *lini*, *D*. *ohnishii,* and *D*. *ogumai*, formed a monophyletic group supported by a high bootstrap value, 96%. This clade (henceforth termed the *lini* clade) formed another, strongly supported (100%) clade with *D. kikkawai* and its siblings, *D. leontia* and *D. bocki*, although the monophyly of the latter three sibling species was not supported. The relationships between the *lini*-*kikkawai* clade, *D. barbarae* (another species sampled from the *kikkawai* complex), and *D. jambulina* of the *jambulina* complex were not resolved. Within the *lini* clade, two distinct subclades, *D*. *ogumai* and *D*. *lini *+* D*. *ohnishii*, were recognized, with high support values, 100% and 98%, respectively. Within the subclade of *D. lini *+* D. ohnishii*, either species did not form a monophyletic branch: the strain DHS315 of *D. lini *branched off first (bootstrap value 93%), followed by the strain MMY326 of *D. ohnishii* (66%), but there was no nucleotide variation in the concerned sequences among the rest strains including those of *D. lini* from Taiwan, Dinghushan (DHS) and Nankunshan (NKS) in Guangdong Province, and MLN24 of *D. ohnishii* from southern Yunnan.

The ancestor of the *lini* clade was estimated to have appeared about 2.23 Mya. Within the *lini *clade, the divergence between *D*. *ogumai* and *D. lini *was estimated to have first occurred 1.42 Mya, and then *D*. *ohnishii* was estimated to have speciated from *D. lini *very recently, at least after 0.17 Mya.

## 4. Discussion

In this study, we tested the applicability of DNA barcoding “species identification” to the *lini* clade consisting of three sibling species, which are morphologically almost indistinguishable [15] but have proved to be more or less reproductively isolated from one another [16, 18]. We took two approaches, the phylogeny-based and character-based methods for DNA barcoding “species identification.” However, neither method succeeded in identifying all these three species. The phylogeny-based method revealed the monophyly of *D. ogumai *and the character-based method found some diagnostic nucleotides for *D. ogumai*, which can be, if not easily, distinguished from the other two species by a few morphological diagnostic characters [15].

However, we failed to get informative sites for DNA barcoding of two very closely related species, *D. lini* and *D. ohnishii*. This provides another evidential case that DNA barcoding is not always effective in species delineation, which has been corroborated in a number of cases of the genus *Drosophila *as a model system [40]. One possible problem is what genes are to be selected for DNA barcoding. Machado and Hey [41] pointed out that the well-established mutual monophyly of two closed related species, *D. pseudoobscura* and *D. persimilis*, was not recovered by phylogeny reconstruction based on nonrecombining molecules (particularly mitochondrial genome), but was strongly supported by that based on recombining molecules (five X-linked loci). The reason for the former is gene introgression between the species [41]. This may or may not be the case between *D. lini* and *D. ohnishii* as well. On the other hand, recombining molecules (inversions regions) may have contributed to the speciation process by affecting the hybrid fitness [42]. So-called speciation genes involved in the pre- and postmating isolations might be good candidate genes for DNA barcoding and, of course, are very important to understand speciation mechanisms of such species at initial speciation. However, DNA barcoding based on such a standard marker as the 648-bp fragment of *COI* in the Barcode of Life project [11, 12] should be a promising tool for nonexperts to easily and rapidly identify most of known species.

The inferred phylogenetic tree based on the concatenated sequences of the three regions did not support the monophyly of the *kikkawai* complex, although the taxon sampling was quite limited, covering only seven out of 12 species of this complex, in this study. Other studies, though under limited taxon sampling as well, suggested nonmonophyly of this species complex [14, 22, 23, 43]. The delimitation of this species complex should be revised on the basis of molecular phylogenetic analyses under more comprehensive taxon sampling. However, the tree strongly supported the *lini* clade comprising the three sibling species, *D. lini*, *D. ohnishii, *and* D. ogumai*, and placed it close to *D. kikkawai* and its sibling species, in consistence with previous studies [14, 20–23].

With respect to the evolution of the *lini* clade, a hypothesis that the premating isolation has evolved through the process of reinforcement in the secondary contact zone between parapatric neighbors, *D*. *ohnishii*/*D. lini* or *D*. *ohnishii*/*D. ogumai*, has been proposed, since the premating isolation is absent between allopatric species, *D. lini* and *D. ogumai* [18]. Based on the phylogeny inferred from the present study and all available biological information from previous studies, we refine or revise the above hypothesis.

The ancestor of the *lini* clade should have derived as a close relative to *D. kikkawai* and/or its sibling species about 2.23 Mya presumably in the subtropics of the Oriental Region. Then, the first speciation event producing *D. ogumai *and *D. lini *may have occurred about 1.42 Mya, and, finally,* D. ohnishii *may have diverged from *D. lini *very recently (at least after 0.17 Mya). This speciation order seems to be congruent with the morphological differentiation among the three species: *D. ogumai *can be distinguished from the other two species by a few diagnostic qualitative characters of the male genitalia and also is most remote from the other two species in terms of morphological distance based on metric characters [15]. In addition, variation in the strength of postmating isolation among the three species seems to be congruent as well with the speculated speciation order: the complete postmating isolation (production of no F1 hybrids) is present between the most diverged species, *D. ogumai *and *D. ohnishii*, while the postmating isolation is partial, producing F1 fertile female but sterile male hybrids, between the other pairs of species [16].

As a cue for the premating isolation caused by female repelling behavior, the frequency of sine song generated by males in their copulatory courtships has strongly been suggested from the evidence that it is different among the three species (significantly lower in *D. ohnishii *than in *D. lini *and *D. ogumai*) in accordance with the modes of sexual isolation between them [18]. Furthermore, wing-cut and playback experiments have provided crucial evidence for that the sine song frequency is used as a sexual cue for mate recognition in the *lini *clade [44, 45]. In light of the inferred phylogeny, it is most parsimonious to consider that the lower frequency sine song has evolved in *D*. *ohnishii*. If so, the first speciation between *D. ogumai *and *D. lini *should have occurred allopatrically, because sympatric or parapatric speciation seems to be inconceivable under the absence of premating isolation. As for the second speciation of *D*. *ohnishii *from *D. lini*, two hypotheses can be conceived with respect to the evolutionary sequence of post- and premating isolations: (1) if the postmating isolation has first evolved, it should have been established between geographically isolated populations as in the first speciation event. Then, the premating isolation, that is, the lower frequency sine song, may have evolved through the process of reinforcement in the secondary contact zone with either neighboring species in *D*. *ohnishii*. Even in the light of the phylogeny inferred from the present study, we cannot determine the secondary contact to which species has promoted this evolution. (2) If the premating isolation has evolved first, the change in frequency of sine song has occurred and fixed in *D*. *ohnishii* as a consequence of adaptation to specific, but unknown, environmental conditions or as a neutral change irrespective of any adaptation and has secondarily come to function, actually or potentially, as a cue for mate recognition by females in this group. If this is the case, *D*. *ohnishii *would have speciated from a small local population, where such (a) mutant gene(s) causing differentiation of sexual character(s) are apt to be fixed, within the range of *D. lini*. However, the mtDNA haplotype polymorphism observed in *D*. *ohnishii *(Figures 1 and 2) seems to be inconsistent with this hypothesis, although it does not rule out the possibility of gene introgression after speciation, especially from southern China (DHS and NKS) populations of *D. lini *to southwestern China (MLN) population of *D*. *ohnishii*.

For the establishment of postmating isolation, another possibility is infection of microorganisms that cause cytoplasmic incompatibility [46–48]. However, Wolbachia infection has never been detected from any strains of the *lini *clade (M. Watada, personal communication). From another aspect, however, there remain large areas lacking distribution data between the ranges of the three species, especially between Xishuangbanna, southern Yunnan (the eastmost population of *D. ohnishii*) and Dinghushan, central Guangdong (the westmost population of *D. lini*). Filling this gap of data will prompt us to revise the hypothesis about the evolution of these two species seemingly having diverged very recently. In addition, studies of speciation genes relating to the post- and premating isolations, especially those underlying the differentiation of sine song frequency, are needed.

## Figures and Tables

**Table 1:** List of experimental strains.

Species	Lines	Collection locality
*D. lini*	3146.1	Taiwan, China
	DHS315	Dinghushan, Guangdong, China
	DHS410	Dinghushan, Guangdong, China
	DHS501	Dinghushan, Guangdong, China
	NKS9212	Nankunshan, Guangdong, China
	NKS9231	Nankunshan, Guangdong, China
	NK9242	Nankunshan, Guangdong, China

*D*. *ohnishii *	MMY309	Pyinoolwin, Myanmar
	MMY326	Pyinoolwin, Myanmar
	MLN24	Menglun, Yunnan, China
	MLN45	Menglun, Yunnan, China

*D.* *ogumai *	RGN3	Yangon, Myanmar
	RGN206	Yangon, Myanmar

*D.* *bocki *	Y163	?
*D.* *leontia *	AO-2	?
*D. kikkawai*	OGS4	?
*D. barbarae*	MLN260	Menglun, Yunnan, China
*D. jambulina*	NH115	?
*D. seguyi*	K2	?

**Table 2:** Target regions and primer sequences in the present study.

Target region	Primer sequence (5′–3′)	Length (bp)
Mitochondrial loci		
*ND2 *	AAGCTACTGGGTTCATACC	926
	ATATTTACAGCTTTGAAGG	
* COI-COII*	ATACCTCGACG(AT)TATTGA	842
	GTTTAAGAAACCAGTACTTG	

Nuclear locus		
*ITS1-ITS2 *	TCCGTAGGTGAACCTGCGG	650
	GTTAGTTTCTTTTCCTC	

Total		2418

**Table 3:** Nucleotides at 20 informative sites in *ND2* sequences of 13 strains of *D. lini *and its sibling species (*D. ohnishii *and *D. ogumai*). Diagnostic nucleotides for DNA barcoding are indicated with an asterisk. *N* is the number of strains sequenced. The positions of nucleotide sites are based on the sequence of *D. obscura *[38]. Polymorphic sites are shown with code letters R (A/G,), Y (T/C), S (C/G), W (A/T), and M (A/C).

Position																						
Species	*N*	*ND2* (sites 34–959)																				Phylogeny
		1	2	3	3	4	4	4	4	5	5	6	6	7	7	7	8	8	9	9	9	
		1	5	4	4	0	1	3	8	4	5	6	6	4	8	9	2	9	0	3	5	
		4	5	2	5	8	1	4	7	3	2	7	9	7	1	6	9	5	0	4	4	

*D*. *lini *	7	R	T	C	A	Y	A	G	S	T	A	W	W	R	T	C	M	C	T	C	C	Non-monophyletic

*D*. *ohnishii *	4	A	T	C	A	C	A	G	S	T	R	A	A	G	T	C	A	C	T	C	C	Non-monophyletic

*D*. *ogumai *	2	G	C*	T*	R	T	G*	A*	G	C*	A	A	A	G	C*	T*	A	T*	C*	T*	T*	Monophyletic

**Table 4:** Nucleotides at 12 informative sites in *COI-COII* sequences of 13 strains of *D. lini* and its sibling species (*D. ohnishii and D. ogumai*). Diagnostic nucleotides for DNA barcoding are indicated with an asterisk. The positions of nucleotide sites are based on the whole length of the *COI* (1536 bp) and *COII* (684 bp) sequences of *D. yakuba* [39]. See Table 3 for further explanations.

Position														
Species	*N*	*COI* (sites 1407–1536)			*COII* (sites 1–639)									Phylogeny
		1407	1485	1503	69	72	231	232	234	399	435	486	570	

*D*. *lini *	7	C	Y	T	T	C	C	T	R	Y	W	A	R	Non-monophyletic
*D*. *ohnishii *	4	C	C	T	T	C	C	Y	A	T	A	A	A	Non-monophyletic
*D*. *ogumai *	2	Y	T	C*	C*	T*	T*	T	A	T	A	G*	A	Monophyletic

**Table 5:** Nucleotides at four informative sites in ITS1-ITS2 sequences of ten strains of *D. lini* and its sibling species (*D. ohnishii* and *D. ogumai*). Diagnostic nucleotides for DNA barcoding are indicated with an asterisk. The positions of nucleotide sites are based on the sequence of *D. ogumai*. Determination of each region in the sequence is based on the whole sequence of *D. simulans* (Z28413), ITS1: 690 bp; 5.8S: 123 bp; ITS2a: 26 bp; 2S: 30 bp; ITS2: 383 bp [26]. The partial or whole sequence of each region was obtained in *D. ogumai* as: ITS1: last 81 bp; 5.8S: 123 bp; ITS2a: 28 bp; 2S: 30 bp; ITS2: first 392 bp. See Table 3 for further explanations.

Position						
Species	*N*	ITS1 (last 81 bp)	ITS2 (first 392 bp)			Pylogeny
		16	307	312–325	326	

*D*. *lini *	6	G	T	GTCAATAATAAAAT	—	Non-monophyletic
*D*. *ohnishii *	2	G	T	GTCAATAATAAAAT/deletion	—	Non-monophyletic
*D*. *ogumai *	2	A*	G*	GTCAATAATAAAAT	T*	Monophyletic
